# Evaluation of the clinical impact of repeat application of hydrogel-forming microneedle array patches

**DOI:** 10.1007/s13346-020-00727-2

**Published:** 2020-02-26

**Authors:** Rehan Al-Kasasbeh, Aaron J. Brady, Aaron J. Courtenay, Eneko Larrañeta, Maelíosa T.C. McCrudden, Donal O’Kane, Stephen Liggett, Ryan F. Donnelly

**Affiliations:** 1 School of Pharmacy, Queen’s University Belfast, 97 Lisburn Road, Belfast, BT9 7BL UK; 2 Belfast Health and Social Care Trust, Belfast City Hospital, 51 Lisburn Road, Belfast, BT9 7AB UK; 3 Belfast Health and Social Care Trust, Royal Victoria Hospital, 274 Grosvenor Road, Belfast, BT12 6BA UK

**Keywords:** Microneedles, Microneedle array patches, Hydrogels, Skin barrier, Biomarkers, Safety, Clinical translation

## Abstract

Hydrogel-forming microneedle array patches (MAPs) have been proposed as viable clinical tools for patient monitoring purposes, providing an alternative to traditional methods of sample acquisition, such as venepuncture and intradermal sampling. They are also undergoing investigation in the management of non-melanoma skin cancers. In contrast to drug or vaccine delivery, when only a small number of MAP applications would be required, hydrogel MAPs utilised for sampling purposes or for tumour eradication would necessitate regular, repeat applications. Therefore, the current study was designed to address one of the key translational aspects of MAP development, namely patient safety. We demonstrate, for the first time in human volunteers, that repeat MAP application and wear does not lead to prolonged skin reactions or prolonged disruption of skin barrier function. Importantly, concentrations of specific systemic biomarkers of inflammation (C-reactive protein (CRP); tumour necrosis factor-α (TNF-α)); infection (interleukin-1β (IL-1β); allergy (immunoglobulin E (IgE)) and immunity (immunoglobulin G (IgG)) were all recorded over the course of this fixed study period. No biomarker concentrations above the normal, documented adult ranges were recorded over the course of the study, indicating that no systemic reactions had been initiated in volunteers. Building upon the results of this study, which serve to highlight the safety of our hydrogel MAP, we are actively working towards CE marking of our MAP technology as a medical device.

## Introduction

**Fig. 1:**
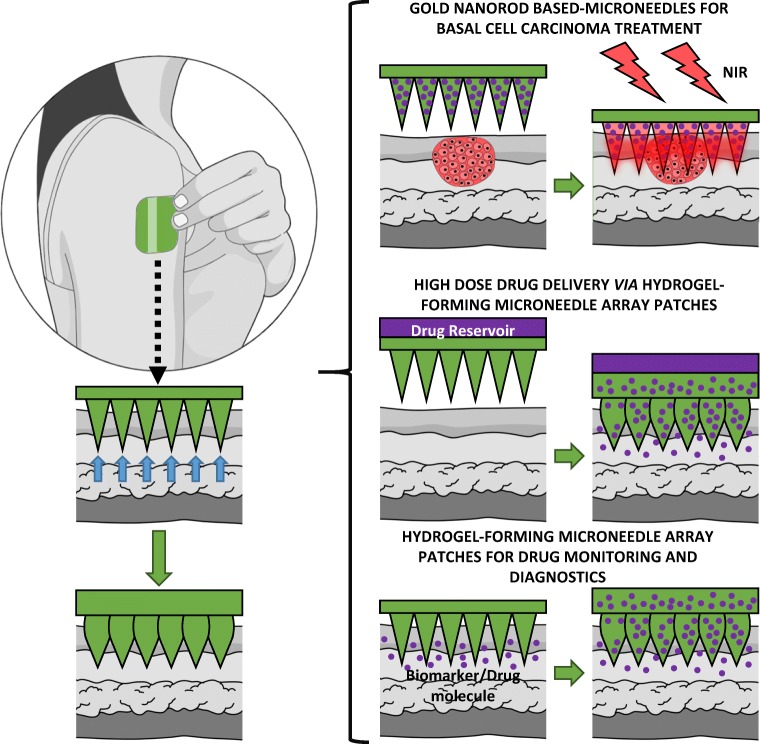
Schematic representations of hydrogel-forming MAP applications

Hydrogel-forming microneedle array patches (MAPs, Fig. 1) are based on arrays of projections less than 1 mm in height perpendicularly arranged upon a flat baseplate, where both the projections and the baseplate are comprised of chemically cross-linked hydrophilic polymer matrices [1]. Upon painless, blood-free, skin insertion, the microneedles quickly swell through uptake of interstitial fluid from the viable skin layers, with the degree of cross-linking determining the rate and extent of fluid uptake [1]. Diffusion of fluid through the swelling needles leads to subsequent swelling of the baseplate. Unlike conventional microneedle delivery systems for drugs or vaccines, the agent to be delivered is not contained with the matrix of hydrogel-forming MAPs. Instead, a separate drug-containing layer is attached to the upper surface of the baseplate. This means that drug dose is not limited to what can be loaded into or coated on to, the microneedles themselves. Upon swelling, a continuous, unblockable conduit between the drug reservoir and the viable skin layers is created. We have made extensive use of this system for transdermal delivery of high doses of both small molecules and macromolecular agents [1], with our data suggesting that appropriately sized patches could be used to administer daily doses of tens or hundreds of milligrammes per day in humans [2]. This creates the possibility for a substantial expansion of the range of types of drug that could be successfully delivered transdermally.

Hydrogel-forming MAPs are removed from skin intact, depositing no measurable amounts of polymer in the viable skin layers [1, 2]. While this is a potential advantage over dissolving microneedle systems in terms of safety, it also opens up the possibility of using such systems for extraction of skin interstitial fluid. Interstitial fluid concentrations often reflect free (unbound and hence pharmacologically active) concentrations of drugs and exogenous substances in plasma. In fact, tissue concentrations are usually more predictive of clinical outcome than total (i.e. free + bound) plasma concentrations [3, 4]. Given the fact that interstitial fluid also contains many endogenous biomarkers of health and disease, minimally invasive sampling without recourse to blood extraction would present a range of important opportunities in diagnostics, patient monitoring and wearable sensors. Indeed, we have successfully used hydrogel-forming MAPs for detection of glucose and caffeine in human volunteers [5]. Intact removal of hydrogel-forming MAPs may also facilitate targeted, minimally invasive, photothermal (PTT) therapy of non-melanoma skin cancers. In PTT, heating the tissue to be destroyed to ≥ 43 °C yields protein denaturation and disruption of the cellular membrane, causing ablation of tumour tissues [6–8]. We have recently loaded hydrogel-forming MAPs with plasmonic gold nanorods. Irradiation of such nanostructures with near-infrared light allows controllable local heating of tissue into which the microneedles are inserted [9, 10]. By using a lower molecular weight cross-linker, these hydrogel-forming MAPs do not swell as extensively. As such, the gold nanorods can be retained within the hydrogel structure during irradiation with near-infrared light to heat and treat the skin lesion. Post-treatment, the microneedles are removed, along with their gold nanorod cargo. This new development presents advantages over systemic injection of the plasmonic agents, where whole body dosing inevitably occurs.

The most common indication of conventional microneedle systems is in vaccine administration [11, 12]. In such cases, only a small number of applications would be necessary to achieve the desired immunological effect. In contrast, hydrogel-forming MAPs used in drug administration or patient diagnosis/monitoring will clearly be an everyday product. Most drug substances must be administered regularly to maintain a therapeutic effect, while patients with ongoing conditions often require repetitive or continuous monitoring. Even when used in management of non-melanoma skin cancers, several applications may be required for tumour eradication. Accordingly, hydrogel-forming MAPs must be shown to not only be capable of being reproducibly inserted into skin on a routine basis but must also have a demonstrated high level of safety.

Our hydrogel-forming MAP technology is based on aqueous blends of poly(methyl vinyl ether-co-maleic acid) and poly(ethylene glycol) [13]. Upon drying in poly(dimethylsiloxane) microneedle moulds, the material is cross-linked by esterification to yield microneedle arrays that are hard in the dry state, but rapidly take up interstitial fluid upon skin insertion [1, 2]. We have shown that such MAPs can be reproducibly inserted into their own skin by human volunteers, even when the patch size is much greater than the 1–2 cm^2^ size typical of microneedle systems [14–16]. We have also shown that the material of these MAPs is biocompatible, has inherent anti-microbial properties and does not support microbial growth [1, 17, 18]. Endotoxin levels are below the US FDA cutoffs for products intended for injection directly into the circulatory or lymphatic systems and these MAPs can be subjected to gamma irradiation without affecting their properties [18]. Recently, we have shown that repeat insertion into mouse skin in vivo, followed by subsequent retention for 24 h did not lead to disturbance in skin appearance, skin barrier function or biomarkers of infection, immunity, inflammation or allergy [19]. While we have already shown on numerous occasions that single applications of these MAPs cause no adverse effects in humans [1, 14, 16, 20, 21], the vital next step in translation of this technology is to now demonstrate, for the first time, the safety of repeat application, with prolonged wear, in human volunteers.

## Materials and methods

### Chemicals

Gantrez® S-97 BF, copolymer of methyl vinyl ether and maleic acid (PMVE/MA) (molecular weight = 1,500,000 Da), was provided by Ashland Speciality Ingredients, Surrey, UK. Poly(ethylene glycol) (PEG, molecular weight = 10,000) was obtained from Sigma Aldrich, Steinheim, Germany. All other chemicals used were of analytical reagent grade.

### MN patch preparation

**Fig. 2:**
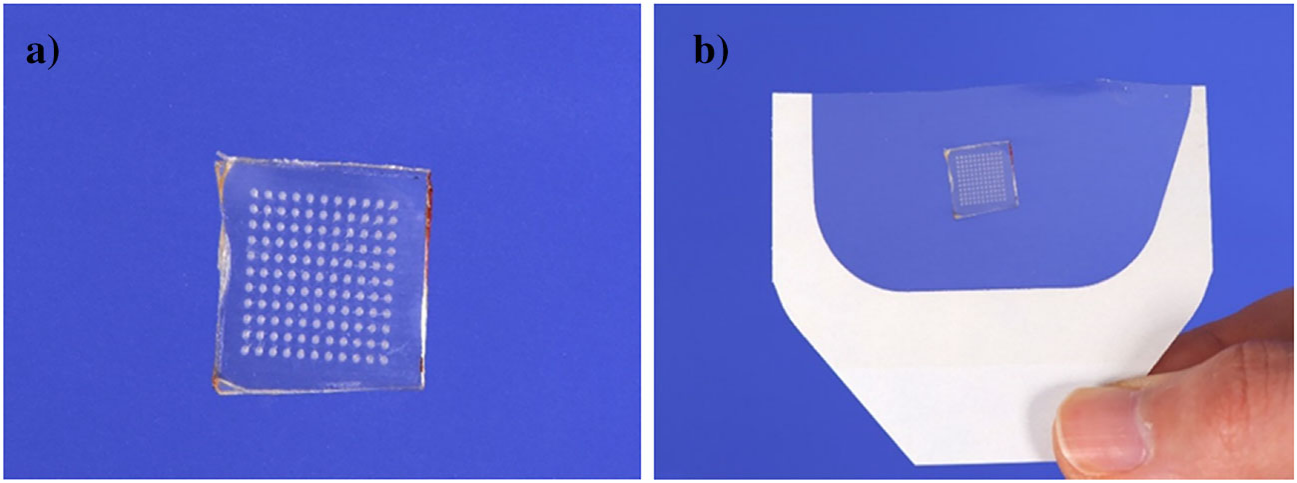
Hydrogel-forming microneedle array patch (MAP) design. Single microneedle array (A); microneedle array attached to the adhesive part of the TegadermTM dressing to form the MAP (B)

Aqueous blends containing 20% w/w PMVE/MA and 7.5% w/w PEG 10,000 Da were utilised to fabricate hydrogel-forming MAPs. The blend (0.5 g) was poured into microneedle moulds (11 rows × 11 rows of needles holes, perpendicular to the base and of conical shape, 600 μm in depth with base width of 300 μm and 300 μm interspacing on a 0.49 cm^2^ area). These moulds were then centrifuged at 2150*g* for 15 min and dried at room temperature for 48 h. The formed microneedle arrays were cross-linked by heating at 80 °C for 24 h and the sidewalls formed by the moulding process removed using a heated blade, as described previously [1, 2]. MAPs were then prepared by attaching the microneedle arrays to a 2 cm × 2 cm piece of Tegaderm™ adhesive dressing (3M, Bracknell, UK) immediately before application to the skin of volunteers, as shown in Fig. 2.

### Volunteer recruitment

Healthy, young, human volunteers (males and females), aged between 24 and 39 years, were recruited to participate in the study. The recruitment was carried out by convenience sampling of the postgraduate student population within the School of Pharmacy. An invitation letter was circulated to the students until sufficient numbers of participants were achieved. If willing to participate, then a participant information leaflet was provided, along with a brief eligibility questionnaire which had to be completed before commencement of the study. Informed consent was obtained in writing from each volunteer before the commencement of the study. All volunteer-related data was anonymised, stored in password-protected files on a fire-walled server and was scheduled for destruction 2 years after completion of the study. Only the researchers directly involved in the study had access to the data. The exclusion criteria were:

- Those who had an active medical condition, such as the common cold, at the time of the study.
- Those with chronic medical conditions or any inflammatory disease.
- Those who had any dermatological conditions, such as acne, eczema or psoriasis.
- Those who had a known allergy or hypersensitivity disorder.

Volunteers were asked to lead their normal life, without taking excessive physical activity while the patches were in place. Participants were also required to keep the MAP application area dry during the period of wear. Furthermore, they were asked not to apply any cosmetic or hygiene-related products to their upper arm during the study period.

### Study protocol

The study was conducted in the Clinical Research Facility Northern Ireland (NICRF) at Belfast City Hospital. Volunteers were asked to arrive at pre-specified times so as to allow MAP application, measurement of transepidermal water loss (TEWL) blood sampling. MAPs were applied by the researcher to a previously marked area on the volunteers’ upper arms. MAPs were applied every day for a period of 5 days, with application alternating from one arm to the other, such that if the upper right arm was the application site on day 1, the upper left arm was the application site on day 2 and so on. Photographic records of the volunteers’ arms aided positioning of the MAPs at approximately the same sites on each occasion.

**Fig. 3:**
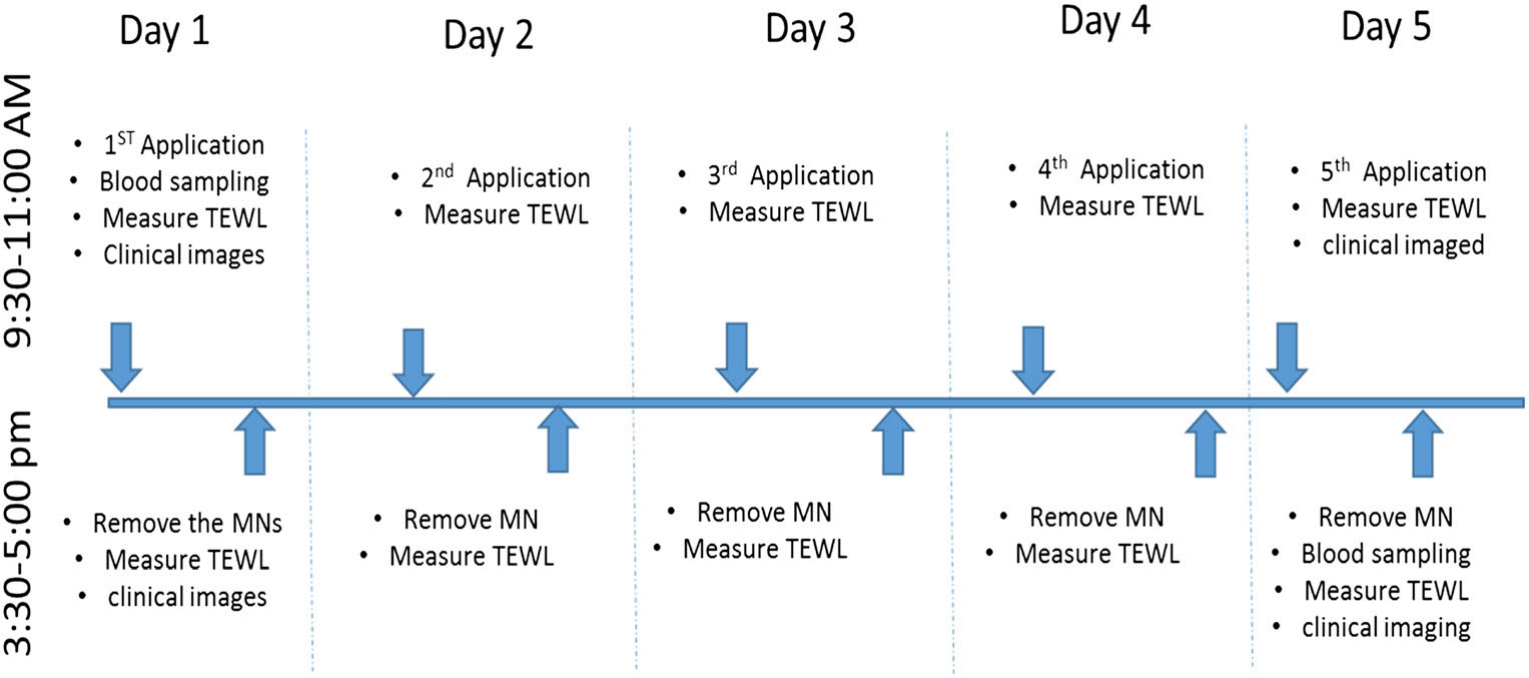
Schematic representation of the study design

### Confirmation of MN insertion, interstitial fluid absorption and swelling using optical coherence tomography and microscopic imaging

Optical coherence tomography (OCT, VivoSight™ Topical Multi-Beam OCT Handheld Probe, Michelson Diagnostics Ltd., Kent, UK) was used to visualise MAP insertion into human skin in vivo. To assess the penetration measurements, quantification was performed using the imaging software ImageJ® (National Institutes of Health, Bethesda, MD, USA). The scale of the image files used was 1.0 pixel = 4.2 μm, thus allowing accurate measurements of penetration depth to be made.

To visualise absorption of interstitial fluids by MAPs, microscope images (Leica EZ4W microscope, Leica, Wetzlar, Germany) of the patches along with digital images of the MAPs collected from volunteers were inspected.

### Blood collection before and after the repeated application of hydrogel-forming MN patches

Blood samples were withdrawn by the stuff nurse. Approximately 10 mL volumes were collected from the volunteers by standard venepuncture technique. The area of the puncture was wiped with an alcohol swap before withdrawal and a sticking plaster (Band-Aid™, Johnson & Johnson, Maidenhead, UK) was applied over the area of blood collection to prevent any further bleeding and to keep the area clean. Blood samples were collected in EDTA-containing blood collection Vacutainer® (Beckton Dickinson, Swindon, UK) tubes. Blood samples were collected at the beginning of the first day and the afternoon of the fifth day, after the end of the last MAP application. Samples were stored at 4 °C until processed. Plasma was collected by centrifugation of collected blood samples at 2000 rpm (2-16k Sigma lab centrifuge, Sigma, Hamburg, Germany) at 4 °C for 15 min. Collected plasma was stored in pre-sterilised Eppendorf tubes and stored at − 80 °C. All samples were recorded on the Human Tissue Act database maintained by the Research and Enterprise Directorate at Queen’s University Belfast according to the policy of the university. All samples were destroyed once the study was completed and, at that time, removed from the database.

### Measurement of skin integrity before and after MN patch application

TEWL measurements have been established as a routine method for evaluating the integrity of skin which has been subjected to either physical or chemical treatment [22, 23]. TEWL was measured in this study to determine the level of disruption to skin barrier function following application of the MN arrays during the study period. The Delfin VapoMeter® (Delfin Technologies Ltd., Kuopio, Finland) is equipped with a closed cylindrical chamber that contains sensors for relative humidity and temperature. The VapoMeter® was used to measure TEWL at the application site (previously marked) pre- and post-application of MAPs every day during the study. Before TEWL measurement, each volunteer was rested for around 5 min to acclimatise to the ambient room temperature. TEWL measurements were taken by carefully resting the Vapometer® probe head perpendicular to the skin. After 20 s, TEWL readings were recorded as the values presented on the digital display unit of the VapoMeter®. TEWL readings were taken before the application, immediately after removal and 18 h after removal of MAPs to determine whether the skin barrier function had returned to normal. On the last day of the study, a control patch composed of Tegaderm™ adhesive dressing without microneedle arrays attached was applied on the opposite arm of the volunteer to evaluate its effect on the TEWL values.

### Clinical scoring of skin irritation and erythema

Clinical scoring was used to determine whether skin irritation or erythema occurred in any of the study volunteers. Clinical scores were based on visual inspection following the scale used for patch (contact allergy) testing according to the guidelines of the International Contact Dermatitis (ICD) Research Group and the North American Contact Dermatitis Group [24]. Clinical photographs of the skin before and after MAP application were captured under controlled lighting conditions by the Medical Photography Team at Belfast City Hospital. Images were scored blindly by an experienced consultant dermatologist. A clinical score for each test site was assigned, using the ICD scores in Table 1.

**Table 1:** ICD clinical scoring scale of patch testing

Score	ICD
0	Negative reaction
+/−	Doubtful reaction; faint erythema only
+	Weak (non-vesicular) positive reaction; erythema infiltration and possibly papules
++	Strong (vesicular) positive reaction; erythema, infiltration, papules, vesicles
+++	Extreme positive reaction; bullous reaction

### ELISA protocols for biomarker quantification in plasma samples

Potential systemic reactions to repeat MAP application were evaluated by quantification of a range of key biomarkers before and after the completion of the application/wear protocol. The biomarkers studied were C-reactive protein (CRP), interleukin 1-β (IL-1β), tumour necrosis factor-α (TNF-α), immunoglobulin G (IgG) and immunoglobulin E (IgE). Commercially available enzyme-linked immunosorbent assay (ELISA) kits were used (Human total IgG Platinum ELISA quantification kit, Affymetrix, Austria; Human IgE Platinum ELISA quantification kit, Affymetrix, Austria; Human CRP instant ELISA quantification kit, eBioscience, Austria; Human TNF-α ultrasensitive ELISA quantification kit, Invitrogen Corporation, USA; Human IL-1β Platinum ELISA quantification kit, Affymetix, Austria), according to the manufacturers’ instructions. Plasma samples were diluted using the sample diluents provided with the kits. Plasma samples for the relevant ELISAs were diluted as follows: CRP, 1:500; total IgG, 1:500,000; IgE, 1:10; IL-1β, 1:2 and TNF-α, 1:2.

The ELISA protocols for the kits were broadly the same. Briefly, 96-well microplates were washed with the requisite washing buffer before addition of the samples. Standards were reconstituted, diluted and added, with the diluted plasma samples, to the plates. Plates were incubated at either room temperature or 37 °C for 1 or 2 h to allow the binding of the biomarker to the primary antibody. The wells on the plates were then washed to remove any unbound antigen. Detection antibody (100 μL) was added to the wells and the microplates were incubated at room temperature with gentle agitation for 1–2 h. The washing step was repeated and aliquots (100 μL) of diluted streptavidin-horse radish peroxidase (HRP) were added to the wells, followed by incubation with shaking for 30 min at room temperature. The washing step was repeated. Substrate (100 μL) was then added to the wells and the colorimetric reaction was terminated by the addition of acid. Sample absorbance readings were recorded at 450 nm on a Microplate spectrophometer (FLOUstar™ Omega, BMG Labtech, Ortenberg, Germany).

### Statistical analysis

Mathematical characterisation of the relationships between the x and y variables in the representative calibration plots was performed using least squares linear regression, following analysis of residuals. Linearity was calculated by linear regression analysis. Means, standard deviation and RSD% were calculated using Microsoft® Excel 2013 (Microsoft Corporation, Redmond, WA, USA). Normality of the data was tested using the Shapiro-Wilk test. Where appropriate, statistical analyses to compare results were performed using a paired *t* test analysis, Wilcoxon signed-rank test and one-way analysis of variance (ANOVA), with post hoc comparisons performed using Tukey’s HSD test. In all cases, *p* < 0.05 denoted statistical significance. The statistical package GraphPad Prism® Version 5.03 (GraphPad Software Inc., San Diego, CA, USA) was used for all statistical analysis.

## Results

### Volunteer recruitment

Eleven healthy volunteers (5 males and 6 females) aged between 24 and 39 years, with no pre-existing skin conditions participated in the study, as presented in Table 2. Volunteers were asked not to apply any cosmetic formulations on their upper arms during the study period. The study was approved by the School of Pharmacy’s Research Ethics Committee at Queen’s University Belfast.

**Table 2:** Demographic information of the human volunteers

Volunteers ID	Gender	Age (years)	Ethnicity
V1	Male	28	Caucasian
V2	Female	28	Caucasian
V3	Male	26	Caucasian
V4	Male	29	Asian
V5	Male	39	Other/Arab
V6	Female	37	Other/Arab
V7	Male	28	Asian/Chinese
V8	Female	29	Caucasian
V9	Female	35	Asian
V10	Female	36	Other/Arab
V11	Female	24	Caucasian

### Skin disruption and recovery measurements

**Fig. 4:**
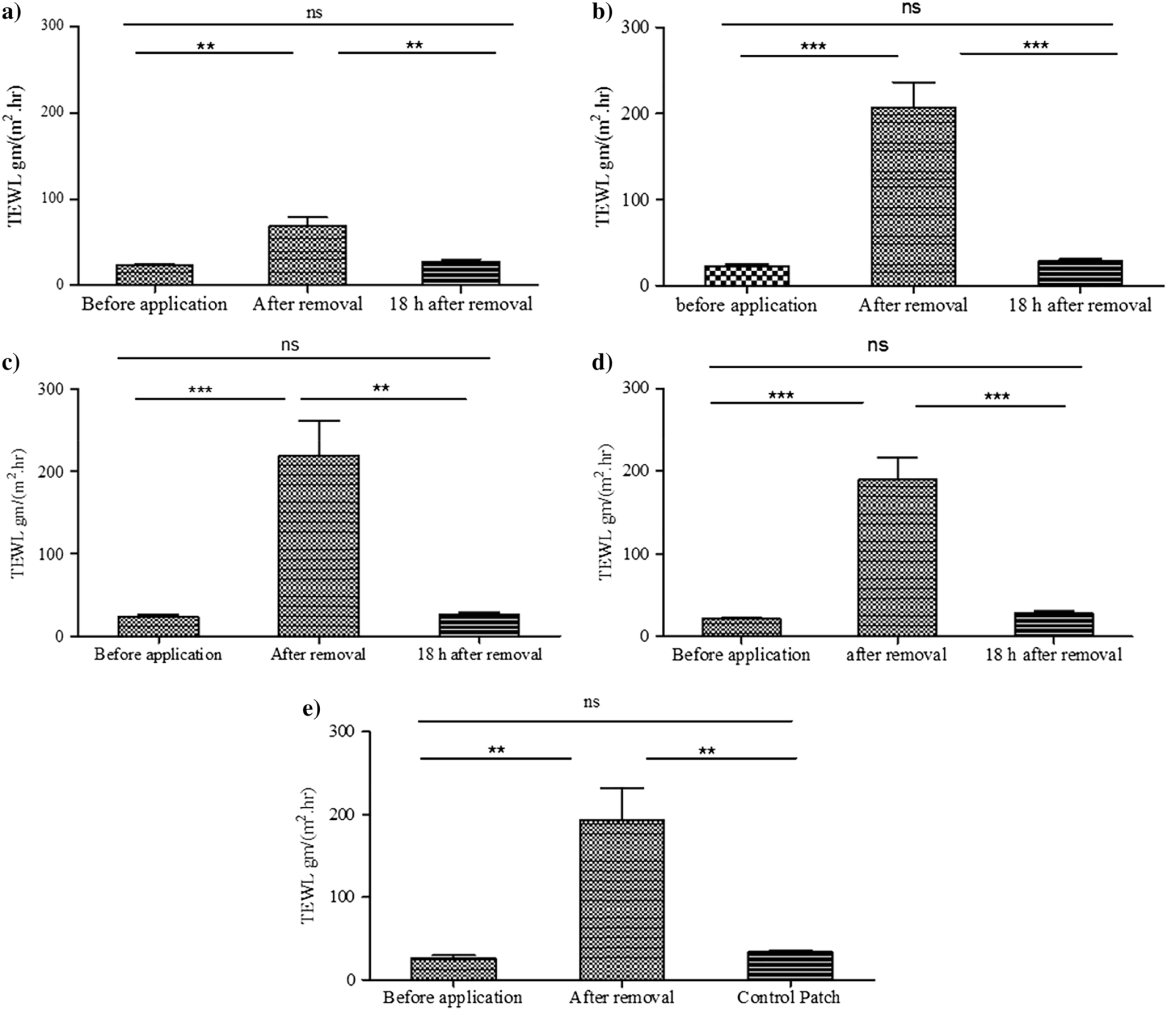
Transepidermal water loss values measured before and after applying MAPs. First day (A), second day (B), third day (C), fourth day (D), fifth day (E). On all days, values increased significantly after MAP removal, but returned to baseline levels 18 h post-patch removal (means + SE, *n* = 11)

TEWL measurements were taken from the site of MAP application for each volunteer before and after MAP application every day during the study. Volunteers were rested for 5 min before the measurement. To confirm complete recovery of skin barrier function, measurements were also taken 18 h after MAP removal. On the last day, a control patch comprised only of Tegaderm™ without microneedles was applied to each volunteer on the opposite upper arm at the same time as the MAP. Prior to MAP application, mean (± SD) TEWL values ranged from 21.15 ± 5.1 to 26.31 ± 10.84 g h^−1^ m^−2^. As expected, when MAPs were removed, the immediately measured TEWL values increased significantly (*p* < 0.05 in all cases) to values between 3 and 10 times the baseline values, as presented in Fig. 4. The increase on the first day, while significant, was not as marked as on subsequent days, possibly due to unmeasurable differences in ambient conditions in what was not a temperature, ventilation or humidity controlled room. On the third day, high standard deviation of 141.1 g h^−1^ m^−2^ was observed as one of the volunteers showed a TEWL value of 495 g h^−1^ m^−2^. These high TEWL values declined, reaching baseline values before the next application, with no significant difference compared with the baseline value on the first day in all cases. Upon removal after 6 h, control patches comprised of Tegaderm™ only, produced TEWL values showed a non-significant (*p* = 0.0659) increase to 33.80 ± 7.16 g h^−1^ m^−2^ of the baseline value of 26.31 ± 10.84 g h^−1^ m^−2^ in the same day. This is expected, as Tegaderm™ dressing application is not thought to have any disrupting effect on the skin barrier function, given that it is a vapour-permeable dressing.

### Confirmation of MAP penetration using OCT and MAP swelling using a digital microscope

**Fig. 5:**
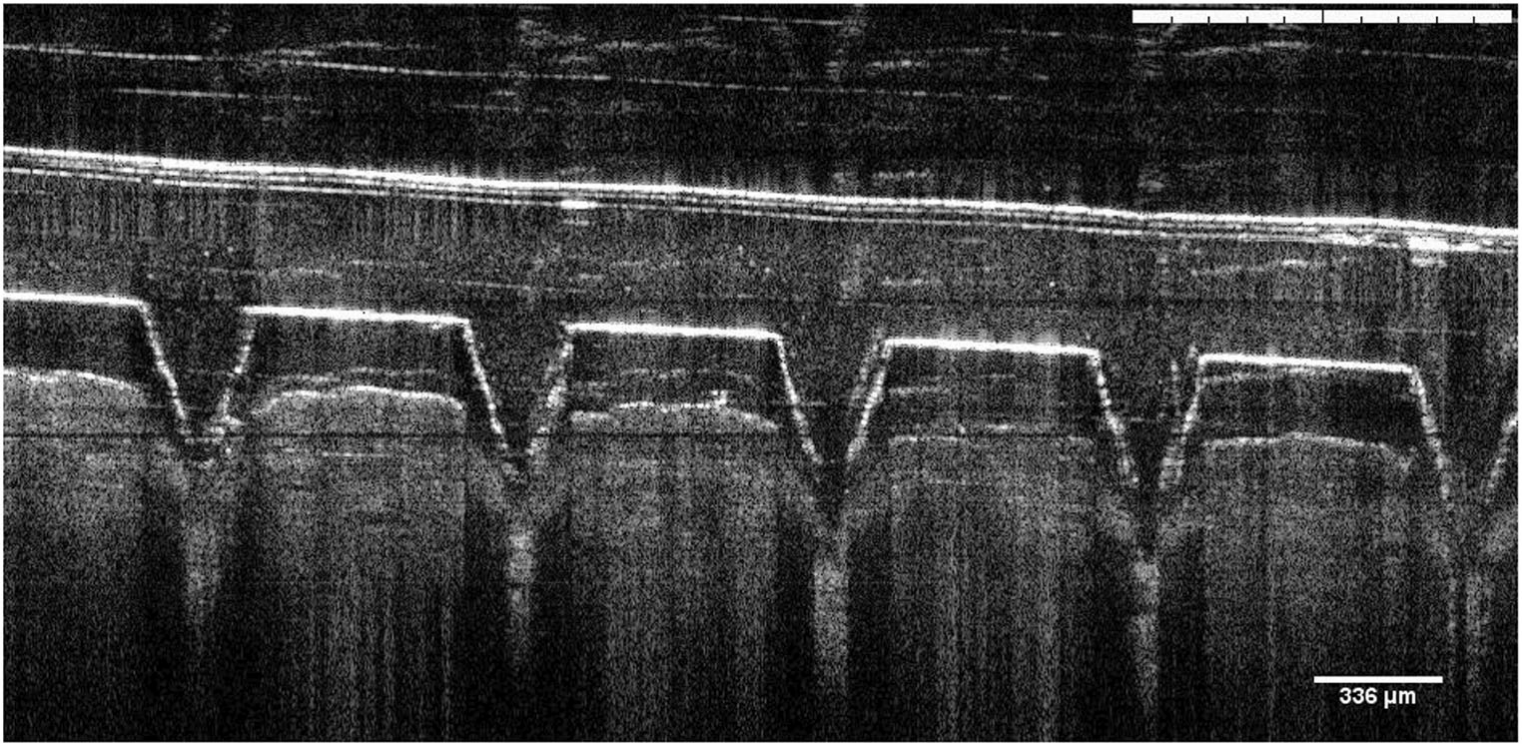
Optical coherence tomography image of MAP immediately after insertion to the forearm of the researcher. It is clear that the MAP was able to create pores and penetrate into the skin upon manual application

To confirm the insertion and penetration of the microneedles into the arms of the volunteers during the study, the researcher applied the same patch to her own forearm on three different occasions before the commencement of the study and recorded the penetration of MN into the skin by OCT imaging. The OCT scan in Fig. 5 was taken immediately after MAP application. It confirms that the microneedles punctured the *stratum corneum* and extended approximately around 300 μm into the skin. The diameter of the pores in the *stratum corneum* induced by the microneedles was approximately 226 μm.

**Fig. 6:**
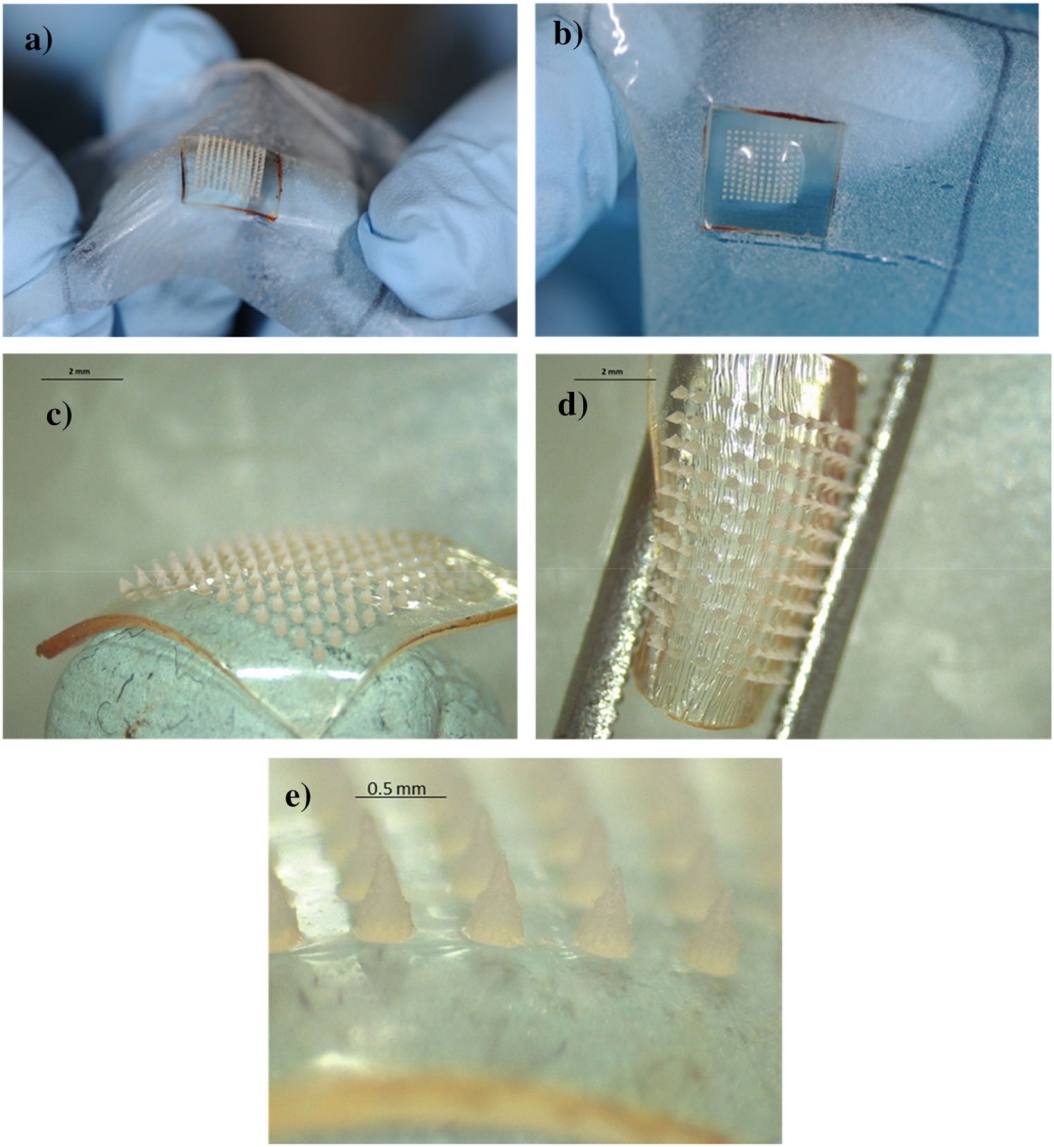
Digital images of MAPs after removal from volunteers’ upper arms. Microneedles and baseplates were flexible and rubbery (A). The array centres showed a higher degree of swelling compared with the baseplates (B). Microscope images of swollen MAPs after removal from a volunteer’s arm. MAPs became elastic as a result of imbibing interstitial fluid from the volunteers (C, D). All of the microneedles of every MAP were removed intact, without any deformation inside the skin (E)

MAPs were visually inspected after removal from volunteers’ arms. Figure 6 A shows the elastic nature of the microneedle array after absorbing interstitial fluid during the 6-h wear period. The arrays showed a curved centre, as a result of the expected higher degree of swelling of the microneedles as compared with the baseplate, as shown in Fig. 6B. Microscope images confirmed that microneedle arrays were flexible and elastic, as shown in Fig. 6C and D. It was noticed that MN arrays remained intact without any loss or damage after removal from the volunteers’ arms, as demonstrated in Fig. 6E.

**Fig. 7:**
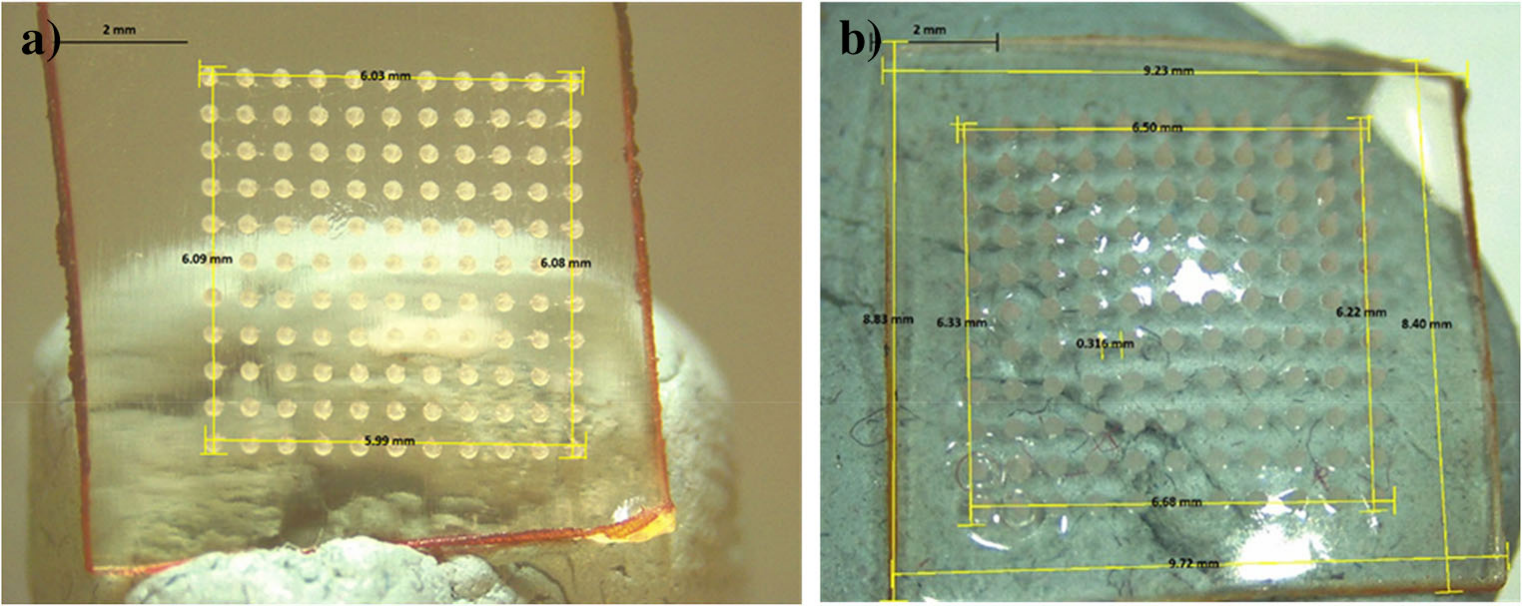
Microscope images of MAPs showing the dimensions of microneedle array (A) before and (B) after application to human volunteers. The area of the array was calculated by measuring the distance between the first and the last microneedle of the first line in the array in mm. Thus, the area was calculated in mm^2^. The application time was 6 h

Microneedle array dimensions were studied before and after wear by calculating the distance between the first microneedle and the last microneedle in the first rows (horizontal and vertical) of the arrays. The mean change in array dimensions post-removal was approximately 12.03 ± 3.43%. Figure 7 shows an exemplar of the measurements before and after the 6-h wear time.

Figure 7 shows exemplar digital images of MAPs at the time of application and after 6 h of wear. It was very apparent that the microneedles remained inserted, despite swelling and the associated dimensional changes of the array, due to the adhesive properties of the Tegaderm™.

### Clinical scoring of skin images before and after MN patch application

**Fig. 8:**
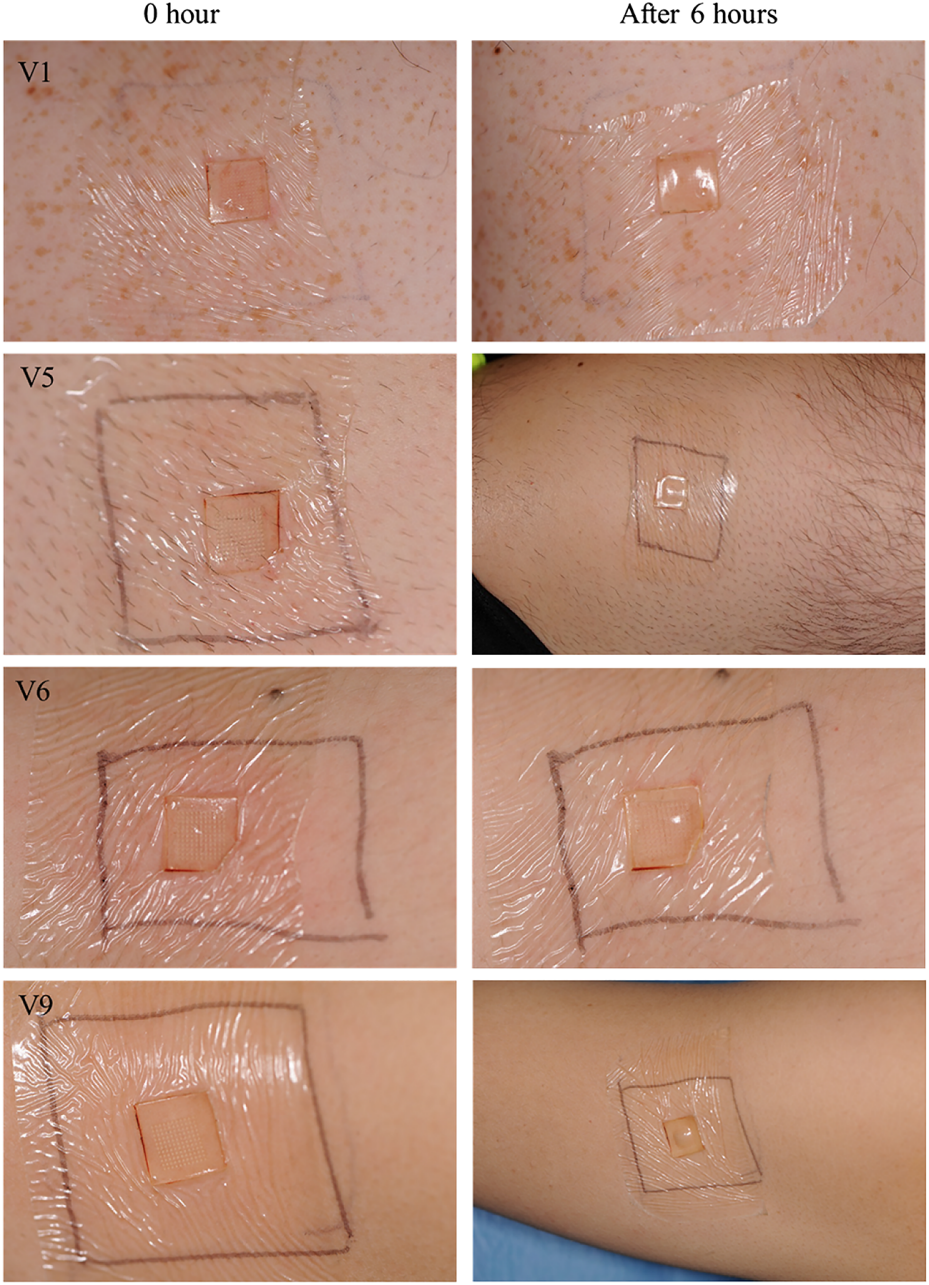
Exemplar images of MAPs immediately upon application and after 6 h of wear. Signs of interstitial fluid absorption were noticed as swelling at the centre of the microneedle arrays in some volunteers. The Tegaderm™ adhesive dressing permitted the microneedles to stay in place over the time of wear

**Fig. 9:**
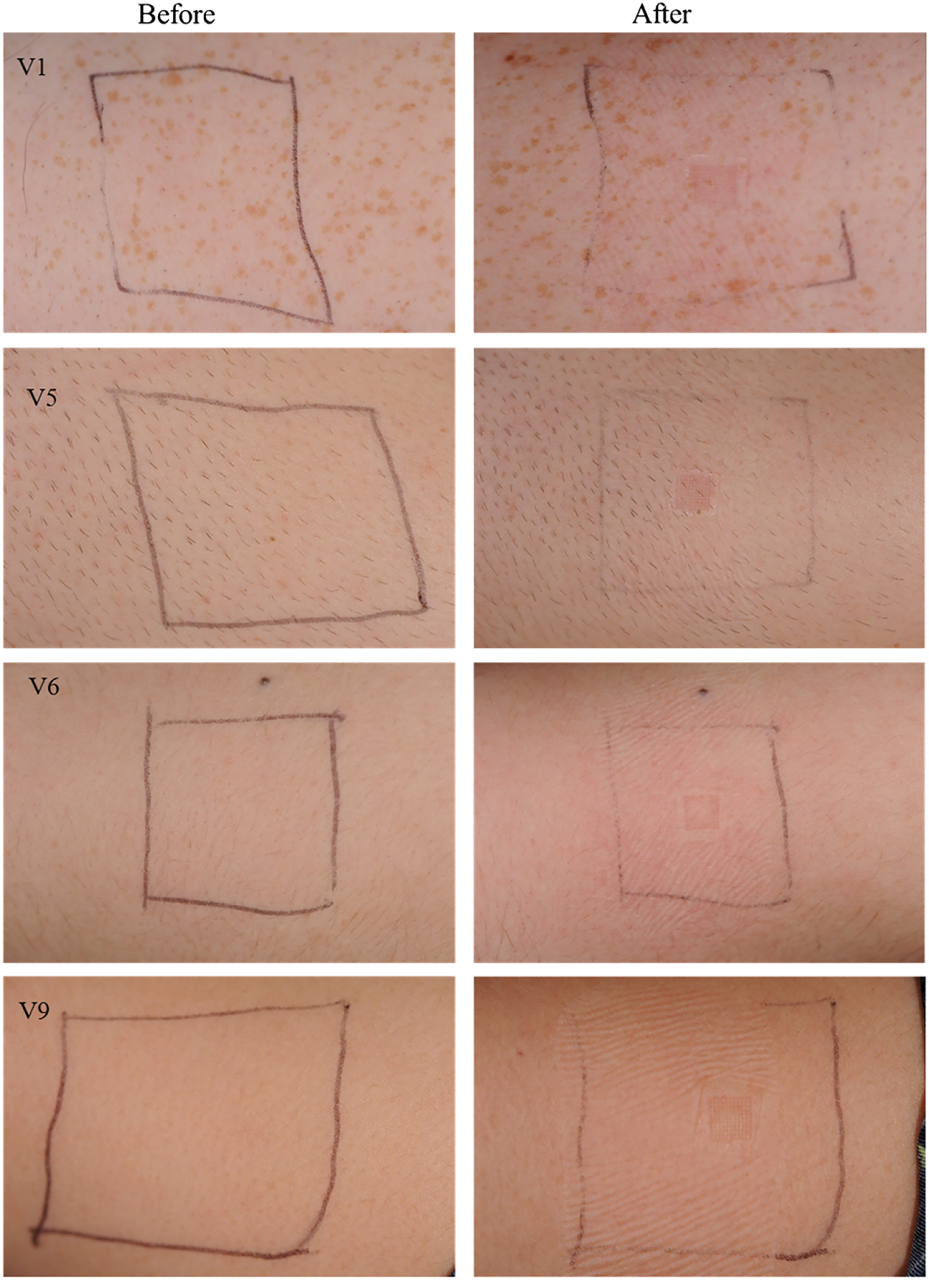
Clinical photographs taken of the upper arm skin of randomly selected volunteers by a medical photographer on the first day of study. Photographs in the left hand column were taken before application of MAPs, while photographs in the right hand column were taken immediately after patch removal (6-h wear time). The area of patch application was marked with a pen

**Fig. 10:**
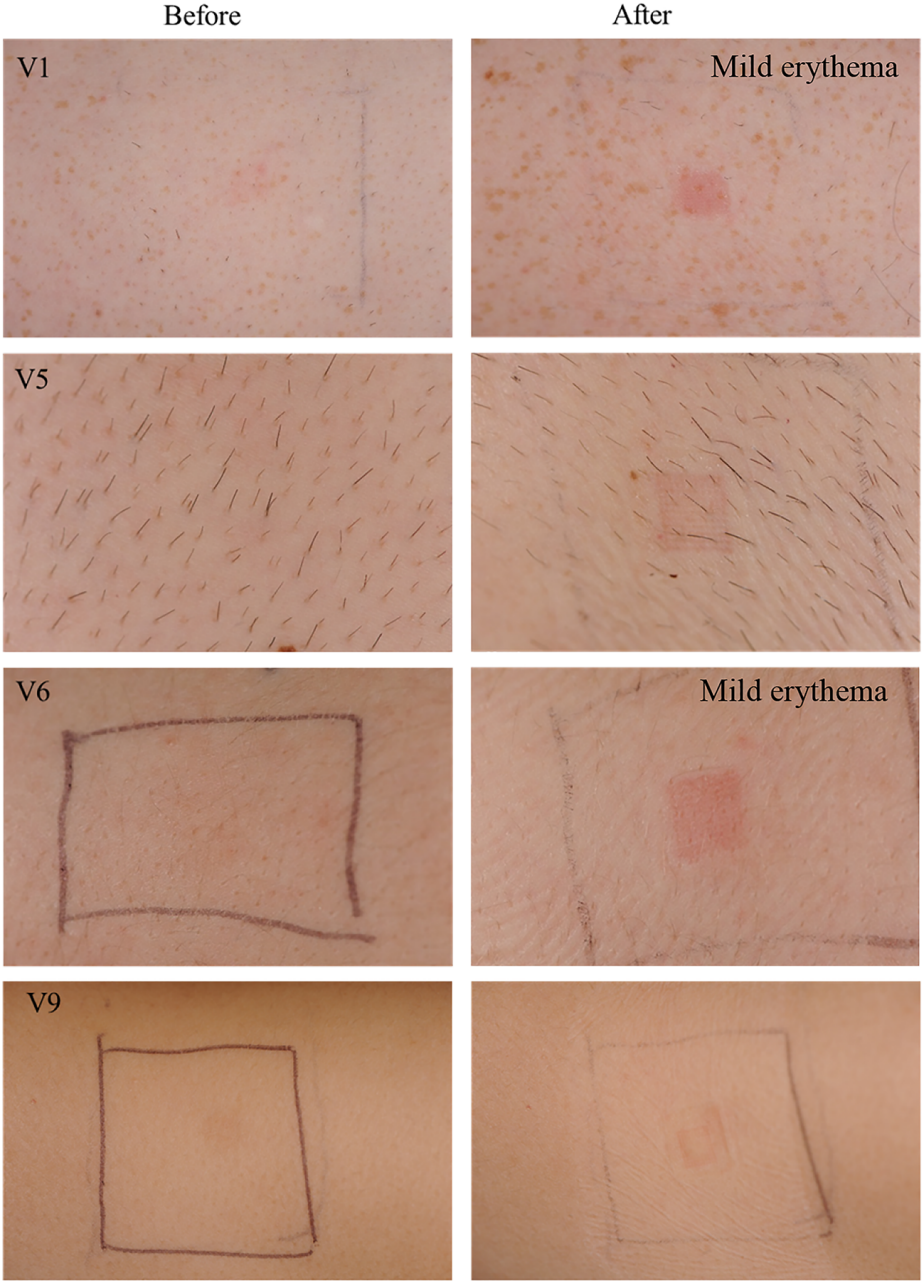
Clinical photographs taken by medical photographer on the fifth day of study. Photographs in the left hand column were taken before application of MAPs, while photographs in the right hand column were taken immediately after patch removal. It is notable that erythema is directly centred to the site of microneedle insertion, rather than in the border area where the adhesive was

**Fig. 11:**
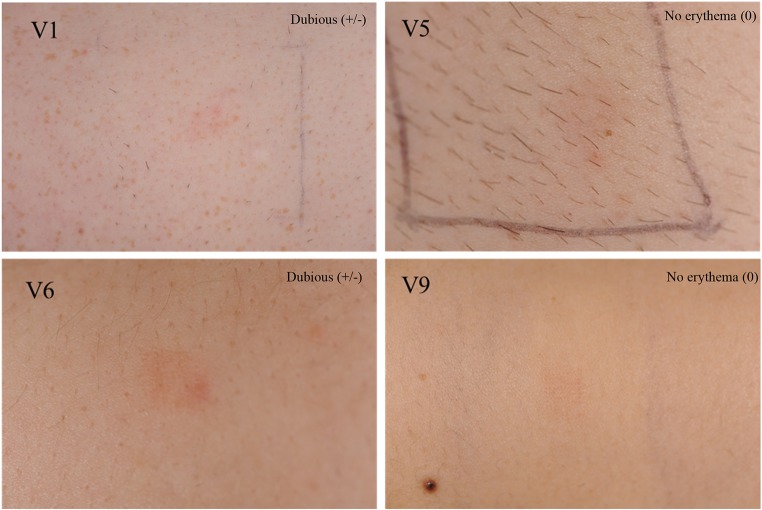
Clinical photographs taken after 18 h of MAP removal. These images were taken on the fifth day of study (i.e. after four MAP applications; two to this approximate site). Erythema degree as assessed by the dermatologist is assigned to each image

Clinical photographs collected at random from four of the volunteers before and after MAP application were scored by visual inspection by an experienced Consultant Dermatologist (DO’K), who was not informed how the skin hat been treated, and an ICD score was assigned to each photograph (Table 3). Figure 8 shows application sites before MAP application and immediately after removal on the first day of study. Figure 9 shows application sites before MAP application and immediately after removal on the fifth day of study. It can be observed that clinical scores immediately after MAP removal ranged between no erythema and mild erythema (Fig. 10). However, as shown in Figs. 11, 18 h post-patch removal, images showed either no erythema or dubious results. In all cases, the erythema score was 0 (no erythema) before the fifth application (approximately 42 h after the third application, thus indicating full disappearance of erythema after that application). Mild erythema was scored in volunteers 1 and 6 after MAP removal on the fifth day, as shown in Fig. 10. Volunteer 9 did not show any erythematous reaction to MAPs after any of the applications, as detailed in Table 3. Slight erythema was visible, however, at the site of MAP application in most of the volunteers, as observed visually, immediately following the removal of the MAPs. It was notable that any erythema observed was directly related to the area of microneedle insertion, rather than the border area where the adhesive Tegaderm™ would have been in direct contact with skin. Unlike any of the other 10 volunteers, one of the volunteers showed a mild erythema at the site of one of the MAP applications after the completion of the study. However, this had fully resolved upon follow-up 7 days later.

**Table 3:** Dermatologist evaluation of medical photographs taken by medical photographer of the upper arm of volunteers after specified MAP applications

Time of photograph capture	Volunteer			
	1 male	5 males	6 females	9 females
Immediately before the first application (baseline)	0	0	0	0
Immediately after the first application	+/−	+/−	+/−	0
18 h after fourth application	+/−	0	+/−	0
Immediately before the fifth application	0	0	0	0
Immediately after the fifth application	+	+/−	+	0

Five distinct biomarkers were quantified using ELISA kits after separating plasma from blood samples. For clarity of data presentation, each volunteer was assigned a distinctive colour in analyses and the same colours were then used for the same individual volunteers in the presentation of all analyses. Calibration curves, constructed according to the manufacturers’ instructions, were used to calculate the levels of biomarkers in the volunteers’ plasma samples. Values were originally calculated in nanogram/millilitre, then corrected to the ranges normally employed for each biomarker by accounting for the dilution factors of the samples.

**Fig. 12:**
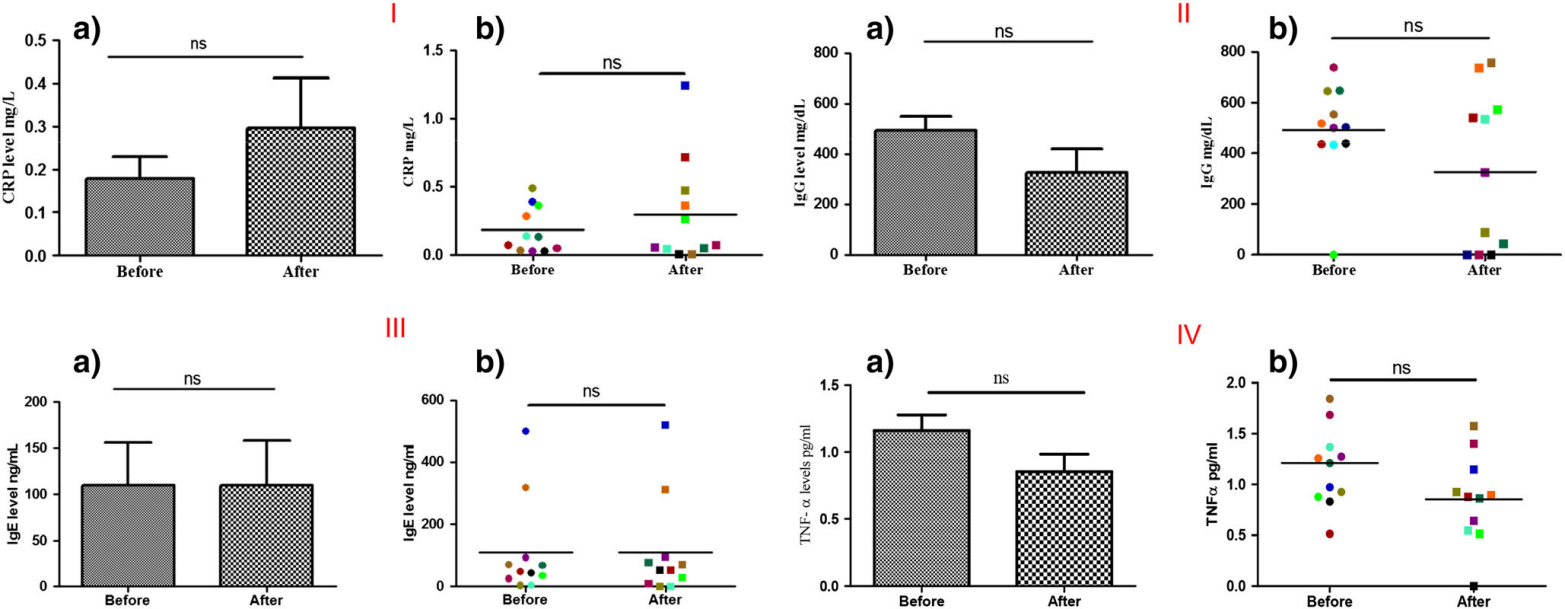
**I**: levels of plasma CRP at the beginning and the end of the study, as determined using ELISA (means ± SE, *n* = 11) (A) and individual volunteer values (B). **II**: levels of plasma IgG at the beginning and the end of the study, as determined using ELISA (means ± SE, *n* = 11) (A) and individual volunteer values (B). **III**: levels of plasma IgE at the beginning and the end of the study, as determined using ELISA (means ± SE, *n* = 11) (A) and individual volunteer values (B). **IV**: levels of plasma TNF-α at the beginning and the end of the study, as determined using ELISA (means ± SE, *n* = 11) (A) and individual volunteer values (B). ns, non-significant

CRP concentrations at the beginning and the end of the study were determined, as presented in Fig. 12 I (A). There was no significant difference in the mean levels of CRP between the beginning and end of the study (*p* = 0.9658). The individual volunteer CRP distribution is shown in Fig. 12 I (B). One volunteer showed a significant change in their CRP plasma concentrations with 0.3 mg/L and 1.24 mg/L measured before and after the study, respectively (marked in blue). However, this individual did not follow the trend of the other participants.

Figure 12 II depicts the levels of total IgG in volunteers’ plasma and indicates no significant difference (*p* = 0.3203) between its level at the beginning and the end of the study. All the measured values were within the normal ranges of IgG in humans.

Levels of IgE in plasma at the beginning and the end of the study are presented in Fig. 12 III. Two volunteers recorded values which did not follow the general trend of the other volunteers (blue and light brown coloured points), but it was noticed that they recorded approximately the same values before and after the study. Hence, the difference between the mean values was not significant (*p* = 1.0000). This indicates that repeat MAP application did not trigger allergic reactions in any of the volunteers.

There was again no significant difference between the levels of TNF-*α* in volunteers’ plasma samples taken at the beginning and the end of the study (*p* = 0.7246), as demonstrated in Fig. 12 IV. All values were within the normal range found in the literature.

Results of the IL-1β ELISA showed that all the volunteers’ plasma levels were below the quantification limit of the kit. Most of the absorbance results for volunteer samples approximated those of the blank values. This indicates that plasma of the volunteers contained undetectable levels of this particular biomarker.

## Discussion

The first patent on microneedle-based delivered systems was filed in the 1970s [25], but it took more than 20 years before the first practical demonstration [26]. Despite regularly being hailed as a delivery system that will revolutionise drug and vaccine delivery and patient monitoring/diagnosis, no true microneedle product is currently marketed for medical indications. However, recent work by specialised microneedles companies, contract pharmaceutical manufacturers and global not for profit agencies has accelerated progress [27–30].

Regulatory approval and market authorisation of any medical device or drug/vaccine product will always be ultimately dependent upon demonstration of safety. In this respect, microneedle systems have performed extremely well in all reported scientific studies and clinical trials, with the only adverse events ever seen being due to inappropriate use in cosmetic treatments [31, 32]. Despite their mechanism of action requiring the puncture of the skin’s protective *stratum corneum* barrier, microneedles have been shown not to cause infection, even when the skin is not pre-cleansed, whether the microneedles themselves were sterilised or not [17, 31, 32]. Importantly, erythema and disruption in skin barrier function caused by the initial skin puncture have also been shown to resolve quickly, generally within less than 24 h for barrier recovery and less than 7 days for erythema [1, 20, 21, 34].

There have been relatively few comprehensive clinical trials of microneedle systems. Those that have been conducted assessed safety only through skin appearance, absence of significant adverse events and general assessments of patient health. Being a vaccination study, the Prausnitz influenza study involved only a single application to each volunteer, as is typical for conventional influenza vaccination [35]. The Zosano zolmitriptan study again involved only a single application [36]. However, the Zosano parathyroid hormone study involved multiple weekly applications [37]. None of these studies reported any adverse event that raised concern. We initially developed hydrogel-forming microneedle systems for high-dose drug delivery and minimally invasive extraction of skin interstitial fluid, with recent work now combining the basic technology platform with plasmonic gold nanorods for photothermal therapy of non-melanoma skin cancers. When used for drug delivery or patient monitoring, this system is likely to be used on a daily basis. Management of neoplastic skin lesions may also require more than a single application. Accordingly, while we have previously demonstrated that our microneedle materials are biocompatible have inherent anti-microbial properties and that the microneedles themselves can reproducibly be inserted into their own skin by human volunteers without adverse events, the critical next step in translation is to comprehensively demonstrate that repeat application is safe. Our initial work in immunocompetent nude mice showed that repeated application and wear of our hydrogel-forming microneedles generated only mild erythema that quickly resolved upon patch removal, as did disruption of skin barrier function, as determined by measuring transepidermal water loss [19]. Key biomarkers were not disturbed by microneedle use. In the present study, our focus has, for the first time, been on repeat application in human volunteers.

To mimic the normal use of a transdermal patch or patient monitoring system, we alternated the site of MAP application over the 5-day study period here. Thus, we studied skin appearance and skin barrier function, as exemplified by TEWL, at two approximate sites on the upper arms of the 11 human volunteers. TEWL readings showed the expected trend, in that they were significantly increased immediately after MAP removal, but then returned to normal when next measured. Within the general trend, TEWL values showed considerable intra- and inter-individual variability between the volunteers on each day of the study. Such variability may be attributable to unmeasured differences in environmental factors such as humidity, temperature or ventilation [38, 39], in addition to some intrinsic factors, such as the physical, thermal or emotional sweating of participants [40], differences in degree of activity or diet over the duration of the study [23]. From a safety standpoint, it is desirable for the created pores to close soon after microneedle removal to prevent permeation of undesired substances or pathogenic microorganisms [41]. The pores are expected to close as a physiological response, due to the skin’s natural repair mechanisms [42]. Upon disruption of the *stratum corneum* barrier, lamellar body secretion is immediately initiated, followed by synthesis of lipids to restore and maintain the barrier [43]. In general, the time taken for this “healing” process depends upon the initial degree of barrier disruption which, in turn, depends on the geometry and dimensions of the microneedle array employed, as well as the length of time the needles array remains inserted in the skin [44]. In the present study, it was shown that skin barrier function had always returned to their normal baseline values when next measured, which was approximately 18 h post-MAP removal. However, according to previous studies performed using our hydrogel-forming microneedle arrays, along with the vast body of evidence on skin barrier recovery following microneedle removal, it is believed that the time required for the skin to reach its normal state would actually be much shorter.

Erythema was investigated to determine potential for local skin irritation, associated with multiple MAP applications. It is crucial to determine the potential irritant effects of microneedles as part of safety assessments of these devices and indeed for any material that is intended to stay in contact with the skin over prolonged periods of time [45]. This is important, as dysfunction of skin barrier, as shown by TEWL studies, is a primary event in development of atopic dermatitis and other ongoing skin disorders [46]. In the present study, clinical scores after treatment were graded from “no erythema” to “severe erythema” using the International Contact Dermatitis Research Group (ICDRG) criteria. In most volunteers, a certain degree of skin erythema was observed immediately after MAP removal. However, this markedly decreased or disappeared when examined 18 h later, indicating that any erythema was short lived. The effects observed are in agreement with other studies that showed any skin irritation caused by microneedle application was short lived, as confirmed by laser Doppler imaging [47] or chromametry [22]. In addition, Wermeling et al. [48] found that erythema caused by microneedle insertion was transient and disappeared after a few hours. Arya et al. [34] showed that mild erythema localised to the site of patch application resolved fully within 7 days. This matches what we saw here in the one volunteer who had mild erythema at one of the MAP application sites beyond the study period, but not for longer than 1 week. Importantly, in the present study, there were no cases where erythema reached moderate or severe scores, even after multiple applications.

It is abundantly clear that skin application of microneedle systems can induce pronounced systemic immune responses, as evidenced by the plethora of published literature on microneedle vaccines [12, 33, 35]. Elevated biomarker levels can also be used to assess, for example skin injury or infection. Indeed, Li et al. [49] used the EIIIA^+^ (526 bp) segment, a very sensitive marker of tissue injury, to show that rat skin pre-treated with super-short 80-μm microneedle arrays and subsequently incubated with an applied culture of *Staphylococcus aureus* was not infected. This study also assessed development of infection by measuring the levels of white blood cells, leukomonocytes and neutrophile granulocyte within the blood. It was demonstrated that there was no significant difference in the populations of three cell types between the microneedle-treated group and an untreated group, confirming that infection did not occur.

We repeatedly applied polymeric microneedle arrays (separately, dissolving and hydrogel-forming microneedle arrays) to immunocompetent nude hairless mice over 3–5 weeks [19]. CRP, total IgG, IL-1β and TNF-α biomarkers were studied, with plasma levels of these biomarkers shown to never be statistically different from untreated controls. The present study represents the first time that biomarkers indicative of safety have been studied in humans following microneedle application.

CRP is a highly conserved plasma protein that is released by the liver into the vascular circulation to activate the complement system. Measuring CRP is perhaps the most practical way to detect and monitor the presence and progress of a systemic inflammatory response due to its rapidity of response, short half-life and relative simplicity of measurement [50]. CRP is stable in serum or plasma and is typically used to diagnose infection and sepsis [51]. Normal CRP levels are between is 0 and 5 mg/L [52]. CRP levels present may be raised dramatically within 72 h due to infection [53]. The data obtained in the present study strongly suggest that no infections occurred, as levels of CRP were within the normal levels with no significant changes noticed over the course of the experimental period. IL-1β is a key member of the interleukin family, which modulates the inflammation caused by bacterial and viral infections [54]. It is mainly produced by the cells of the innate immune system. However, some of the epithelial cells of the skin can express IL-1β, which explains the expression of this factor in psoriatic disease states [55]. Samples used in the IL-1β ELISA were quantified in diluted and undiluted states, in order to ensure that all levels, including those of neat plasma samples, were below the detection limits of the system employed. Results indicated that all samples were below the detection limits. The detection limit was 0.3 pg/mL, according to the manufacturer’s instructions. TNF-α is released by different types of immune cells to mediate the events involved in inflammation and infection. Thus, it plays a crucial role in the defence against fungi, bacteria and parasites by regulating the proliferation of T-cells [56]. TNF-α levels were below 2 pg/mL in all volunteers, which agreed with the normal levels found in the literature, in which the range was between 0.0 and 32.5 pg/mL [57]. IgG is one of the most abundant proteins in human serum, accounting for about 10–20% of plasma proteins. It is the major class of the five classes of immunoglobulins in human beings, as it accounts for about 75% of the total immunoglobulins in the plasma of healthy individuals [58]. IgG antibodies have a relatively high affinity and can persist in the circulation for a long time [59]. Total IgG levels were found to be within the normal human adult range of 639–1.349 mg/dL [60]. It should be noted, of course, that IgG levels can often take weeks or months to peak, with the relatively short duration of the present study precluding determination of any delayed rise, however, unlikely. IgE is a type of antibody synthesised by plasma cells in mammals. It is identified as the key molecule in mediating what are described as type-1 hypersensitivity reactions (allergic asthma, allergic rhinitis, food allergy, atopic dermatitis, some forms of drug allergy and insect sting allergy) [61]. It is the least expressed immunoglobulin compared with the other types of immunoglobulins [62]. Increased levels of IgE in the blood trigger a cascade of events that cause allergic contact urticaria, a hypersensitivity reaction manifested by immediate but transient localised swelling and redness that occurs in the skin after direct contact with an offending substance [63]. In the present study, all volunteers showed IgE levels below 500 ng/mL. Literature studies have stated that IgE is normally present in plasma at a concentration of less than 1 μg/mL [64]. Accordingly, an allergic reaction was not seen in this study.

This study serves to demonstrate the initial safety of prolonged hydrogel-forming MAP application by assessing basic immunological markers known to be elevated in both skin and systemic immune responses, infection, inflammation and allergy. It is worth noting that further, more detailed, immunochemical analysis is now needed to evaluate the long-term serological impact, if any, of repeated and prolonged application of hydrogel-forming MAPs to the skin and indeed to the body when MAPs are used clinically or periods of weeks and months.

## Conclusion

The present study complements an already strong body of evidence on the safety of hydrogel-forming microneedle array technology. We have shown, for the first time, in human volunteers that repeat application and wear of these microneedles does not cause any prolonged skin reactions or disruption of skin barrier function and does not disturb the normal balance of key systemic biomarkers of inflammation, infection, allergy or immunity over a fixed study period.

Conclusions about the impact of repeat application/wear of microneedles prepared from other materials cannot be appropriately drawn from this work, however. We are currently working towards CE marking of our hydrogel-forming microneedle array technology as a medical device. Development of the technology as a drug or combination product will further require demonstration that intradermal administration of a drug of interest does not cause any adverse event not seen with conventional routes of administration, in addition to showing acceptable pharmacokinetics and pharmacodynamics. This will need to be done on a case-by-case basis.
